# Bronchoscopy in the Pediatric Cardiovascular Patient with Persistent Respiratory Pathology

**DOI:** 10.3390/jcm14186606

**Published:** 2025-09-19

**Authors:** Ana-Belen Ariza-Jimenez, Delia Valverde Montoro, Pilar Caro Aguilera, Estela Perez Ruiz, Francisco Javier Perez Frias

**Affiliations:** 1 Instituto Maimonides de Investigación Biomedica de Cordoba, Hospital Universitario Reina Sofia (Córdoba), Universidad de Cordoba, 14004 Cordoba, Spain; 2 Unidad de Cuidados Intensivos Pediatricos, Hospital Regional Universitario de Málaga (Materno-Infantil), 29011 Málaga, Spain; delia__86@hotmail.com; 3 Neumología Infantil, Hospital Regional Universitario de Málaga (Materno-Infantil), 29011 Málaga, Spain; pimacaro@yahoo.es (P.C.A.); estelaperezruiz@yahoo.es (E.P.R.); fjperez@uma.es (F.J.P.F.); 4 Universidad de Málaga, 29011 Málaga, Spain

**Keywords:** bronchoscopy, congenital heart, airway malformations, heart postoperative complications, extubation failure

## Abstract

**Background:** Patients with congenital heart disease can associate malformations. The most frequent complications are those related to the airways, which produce prolonged cardiovascular postoperative. **Objectives**: Describe pathology, bronchoscopy indications, and findings in patients with heart pathology and persistent breath failure to improve prognosis and determine an early treatment. **Methods**: Retrospective descriptive study of bronchoscopies performed during 24 years in pediatric patients with congenital heart disease with surgery indication and persistent respiratory symptomatology. **Results:** We performed 199 fibrobronchoscopies in 144 patients, with an average of 1.4 fibrobronchoscopies per patient. A total of 58% were male. The mean age was 27.5 months (5 days–13 years). Valvular disease was the most frequent congenital heart disease, followed by the transposition of large vessels. The most frequent indications were stridor (42.7%) and persistent atelectasis (24.6%), followed by extubation failure (12.4%) and pump output (6.2%). The majority of the findings were found in the upper airway (56%), with a clear predominance of malacias (32%), while in the lower airway, extrinsic compression was highlighted (42%). **Conclusions**: Flexible fiberoptic bronchoscopy is a useful and rapid method for the diagnosis of airway malformations associated with congenital heart diseases that may have a relevant role in its management and prognosis.

## 1. Introduction

In children, airway compression is usually of vascular origin, being less frequently caused by respiratory pathology, tumors, heart disease, cysts, or abscesses [1]. Considering the relationship of the trachea and bronchi with respect to the thoracic blood vessels, any variation in this anatomy could produce airway compression [2].

Despite this, vascular tracheobronchial compression is uncommon (26%) and underrecognized [3,4,5]. However, it is a significant cause of morbidity with variable, nonspecific symptoms that lead to underdiagnosis and delayed diagnosis [1,2,3,4,5,6,7,8,9,10]. In fact, central airway stenosis following congenital heart malformation surgery is a rare but significant cause of postoperative weaning failure [11], given that airway anomalies increase risk of morbidity and mortality in postoperative pediatric patients with congenital heart disease [12].

Tracheobronchial compression is associated with laryngomalacia and expiratory stridor in a high number of children, so its study would be interesting [2,3,8,10,13]. Furthermore, it is related to increases in the risk of respiratory tract infections, hindered recovery, life-threatening, and long-term cardiac disease, which leads to poor prognosis and increased morbidity, characterized by a heightened risk of heart failure, arrhythmias, and premature death [14].

Airway obstruction may result from an abnormal relationship between the tracheobronchial tree and vascular structures, or the result of extrinsic compression by dilated pulmonary arteries, enlarged left ventricle, massive cardiomegaly, or intraluminal bronchial obstruction [4,5,15,16,17].

Depending on the type of anomaly, these cause pulsatile compression in specific areas of the airway:

Innominate artery: middle third of the trachea.

Double aortic arch: anterior and posterior distal right trachea.

Anomalous pulmonary artery origin: right distal trachea and right bronchus.

Failure of diagnosis and/or failure of treatment can result in progressive respiratory deterioration, and even death due to complete obstruction, anoxia, apnea, and bradycardia [3,7,10,18].

Furthermore, tracheomalacia and tracheobronchomalacia may be primary abnormalities of the large airways or associated with congenital and acquired conditions. Clinical presentation includes early-onset stridor or fixed wheeze, recurrent infections, brassy cough, and even near-death attacks, depending on the site and severity of the lesion. Diagnosis is usually made by flexible bronchoscopy in a free-breathing child but may also be shown by other dynamic imaging techniques such as low-contrast volume bronchography, computed tomography, or magnetic resonance imaging. According to the European Respiratory Society (ERB), they can be described as mild (50–75% loss of cross-sectional area), moderate (75–90% reduction), or severe (>90% reduction). Management may be medical or surgical, depending on the nature and severity of the lesions. Parents of children with tracheobronchomalacia report diagnostic delays and anxieties about how to manage their child’s condition and want more information [19].

Flexible bronchoscopy (FB) is a rapid, accurate, safe, and well-tolerated diagnostic method that makes an important contribution in this field by recognizing lesions, especially those that compress, and identifying their location and degree of stenosis produced [3,5,8,11,14,20,21,22]. In a patient under mild sedation, FB is the only method to evaluate the functional component of malacia. Furthermore, it optimizes perioperative management, as it can detect airway alterations that may cause potential complications [11,14,20]. For this reason, all children diagnosed by another method, such as angiography or barium esophagogram, should be studied via bronchoscopy. In the case of anterior compression diagnosed with bronchoscopy, it would also be interesting to associate a barium esophagogram to predict whether there would be worsening of obstruction after surgery [3,4,9]. To evaluate peripheral structures, it is necessary to combine a computerized tomography scan (CT) with contrast, which also provides more information on the nature of the injury and has greater diagnostic sensitivity [5,7,8,13,14,15,23,24].

During corrective surgery, FB ensures adequate manipulation [2,8,11]. Patients with vascular and cardiac defects also have a high incidence of intraoperative and postoperative airway compression, particularly in end-to-side anastomoses. This, combined with the high risk of morbidity and mortality associated with cardiovascular surgery with tracheobronchial compression, makes intraoperative FB a useful tool for identifying and resolving problems [4,11,24,25]. Intraoperative FB has been used as part of the surgical technique for broncho-guided aortopexy, demonstrating postoperative airway improvement [11,16,17,24,26].

Once diseases such as gastroesophageal reflux, immunodeficiencies, and immotile cilium syndrome have been ruled out, the diagnosis is confirmed. In half of cases, depending on the type and degree of compression, surgical correction is indicated, which is often effective [2,3,7]. Some of the surgical indications are complete vascular rings, anomalous origin of the pulmonary artery, double aortic arch, and any combination of symptoms that significantly influence quality of life [2,3,27].

Airway stents are limited by availability and size and can be placed through FB [11,18].

Persistent airway obstruction after surgery may be secondary to residual compression, malacia, or intrinsic airway lesions [4,6,27].

Factors associated with susceptibility to respiratory disease recurrence include airway stenosis greater than 75%, improvement of less than 50% in diameter after correction, severe preoperative respiratory failure, and male sex [28].

Its underdiagnosis, together with significant morbidity and associated mortality of between 3 and 21%, with 10% of post-surgical sequelae, make this pathology a topic worthy of study in order to make an early diagnosis that minimizes these risks [4,6,9]. This would justify carrying out this descriptive study to provide information that guides what to look for in the indication of bronchoscopy and enable early diagnosis.

Objectives: To determine whether routine and protocol-based bronchoscopies should be performed in all patients with congenital heart disease to identify associated respiratory pathology early and improve prognosis through early treatment.

## 2. Materials and Methods

We show a retrospective, descriptive study, using a database review, of bronchoscopies performed over a 24-year period (1991–2015) in patients with persistent respiratory symptoms associated with congenital heart disease who required surgical intervention.

### 2.1. Center

All patients were from the pediatric pulmonology service of a tertiary children’s hospital, which treats children under 14 years of age from both rural and urban settings, as it is the only referral hospital in the area.

### 2.2. Inclusion Criteria

Children between the ages of 0 and 14 admitted to our center who required BP in the context of cardiovascular disease requiring intervention.

### 2.3. Methodology

Once the patient’s history has been reviewed, in the case of non-urgent interventions requiring immediate on-site care, the bronchoscopy is scheduled using a flexible bronchoscope. This procedure could be performed depending on the patient’s condition and situation in the operating room or pediatric intensive care unit.

Despite being a public university hospital, all pediatric bronchoscopy procedures were performed by the pediatric pulmonology department by two pediatric pulmonologists with expertise in bronchoscopy, who did not change during the study period.

All bronchoscopy procedures were performed under the supervision of the anesthesiologist with IV anesthesia, benzodiazepines, and/or muscle relaxants, in addition to continuous vital signs monitoring. Intubation was not required in all cases, except for those requiring a mask or those with tracheostomies. Once the procedure was performed, the examination and, if necessary, treatment, were recorded in the department’s classified Excel record, for which diagnostic coding is no longer used. Using this record, a retrospective descriptive review of all patients was conducted.

## 3. Results

Along for 24 years, 1591 FB were performed by the pediatric pulmonology service, of which 199 were performed on patients with congenital heart disease undergoing surgery, with an average of 1.4 bronchoscopies per patient. More than one bronchoscopy was required in cases where the patient was not in optimal condition for diagnostic bronchoscopy, such as suspected upper airway abnormalities in bronchoscopies performed with an endotracheal tube or mask, and in cases where a single therapeutic intervention was not sufficient, as in some cases of stenosis or mucous plugging. This represents approximately 8–9 FB per year in patients with cardiovascular disease.

An amount of 95/199 (47.7%) were performed in the operating room and 104/199 (52.3%) in the pediatric intensive care unit. A total of 144 children were evaluated, of whom 80/144 (55.6%) were boys. The mean age was 27.5 months (range: 0.16–158 months). Eleven were performed in neonates (5.5%), 102 in children under one year of age (51%), and 86 in children over one year of age (43%).

The entry route was nasal in 57.78% of cases, endotracheal tube in 26.66%, laryngeal mask in 9.33%, tracheostomy in 5.33%, and CPAP mask in 0.89%.

The congenital heart defects presented are shown in Table 1.

An amount of 107/199 (53.8%) FB were performed in children with non-cyanosing congenital heart disease and 92/199 (46%) with cyanosing congenital heart disease.

Patients requiring more FB were those with tetralogy of Fallot, with 1.8 FB per patient.

An amount of 29/144 (20%) patients had associated pathology because the heart disease was part of a polymalformative syndrome, with Down syndrome being the most common association (18/144) (12.5%).

Among the newborn group, we found 60.87% of infants with cyanotic congenital heart disease diagnosed at birth. However, fiberoptic bronchoscopy was performed at a mean age of 24 days. Among these patients, 13% were syndromic, and there was a 17.4% extubation failure rate.

Regarding infants under 1 year of age, 54.84% had cyanotic congenital heart disease, and the mean age at fiberoptic bronchoscopy was 5.29 months. In total, 32.26% had associated syndromes, and 12.9% of cases failed extubation.

In both groups, oxygen therapy was required in 100% of cases.

There is not any statistically significant relation (p0.1) between the delayed diagnosis and the age of these patients.

The FB findings are shown in Table 2, and the indications for FB for the whole sample are shown in Table 3.

Fiberoptic bronchoscopies performed for intraoperative pump failure occurred in the majority of cases (66.6%) in patients with Down syndrome and AV canal, followed closely by those with transposition of the great vessels.

In all cases, they contributed significantly to clinical decision-making regarding the patient.

In total, 23.66% of the procedures were diagnostic and therapeutic, while the remainder were solely diagnostic.

It is worth noting that 100% of the findings required treatment, whether physical therapy, mucus removal, positive ventilation, administration of anti-inflammatory drugs, instillation of corticosteroids, or surgical or bronchoscopic intervention. Regarding those who required surgery, 30% required airway dilation, 42% required surgical correction of the structures compressing the airway as well as stent placement, and 6.9% required surgical correction of airway fistulas. All of this allowed patients to be classified by risk and to receive good results and positive outcomes during follow-up in the outpatient clinic. In fact, sample survival was 100%.

It is noteworthy that there were no statistically significant differences between the bronchoscopic findings of cyanosing and non-cyanosing heart disease.

However, 43.75% of patients in whom extrinsic compression was detected by fiberoptic bronchoscopy had cyanotic congenital heart disease (*p* = 0.015).

Finally, while some FB combined both lower and upper airway pathologies, 8.5% of the bronchoscopies yielded no findings.

## 4. Discussion

Airway complications are closely linked to poor outcomes in cardiovascular patients undergoing surgery [11,12]. Bandla et al. indicate that the average length of stay and extubation failure are caused in 60% of cases by airway abnormalities, pulmonary hypertension, and phrenic nerve palsy [27]. Nayak et al. find airway narrowing in 50% of cardiovascular patients undergoing surgery with extubation failure [29]. Wu et al. discover 22.6% of airway anomalies in patients undergoing surgery for congenital heart disease, including structural lesions in 78.9% and dynamic problems in 47.0%. They associate these findings of airway anomalies with longer postoperative intubation duration and greater hazard of intermediate mortality [12].

Given that this is a topic that has rarely been discussed in general pediatric settings, and given its importance to patient outcome, prognosis, and healthcare costs, our study is justified. It presents 199 FB performed over 24 years, while the biggest cohort published to date shows 185 FB performed over 3 years [12], and the second most complete cohort published to date presents 104 fiberoptic bronchoscopies performed over 12 years [21].

Therefore, the strengths of this study include the number of procedures reviewed (*n* = 199), the long study period (24 years), and the fact that most similar studies have been conducted in different populations (mostly Anglo-Saxon and Asian). Likewise, little has been published on this topic in children. It is noteworthy that all studies on this topic used FB for diagnosis and treatment of these patients, as did ours, while Gaafar AH et al. report using rigid bronchoscopy [23].

Most of the literature only addresses the lower airway [8,9,11,13,20,22,23,24,26,27,28,29,30,31,32], whereas we present a review of both upper and lower airway locations.

On the other hand, the limitations of our study are due to the retrospective nature of the study, which may lead to information bias, and to the fact that bronchoscopies performed using an endotracheal tube or a laryngeal air mask provided limited visualization of the upper airway, resulting in more than one bronchoscopy per patient. Furthermore, the diagnosis of malacia depends, in part, on the patient’s sedation level, as it is more accurate to assess airway dynamics. Therefore, those diagnosed intraoperatively with muscle relaxant and mild anesthesia may have been affected, necessitating a repeat bronchoscopy during follow-up. In addition, the severity of the malacias according to the ERB classification was not recorded during the study.

Vascular compression of the airway is present in 1–2% of these patients [16]. According to the literature, the vascular anomalies found, from most to least frequently, are double aortic arch, innominate artery, brachiocephalic trunk, vascular rings, right aorta with arterial ligament or diverticulum, anomalous origin of the pulmonary artery, and dextrocardias with double vena cava, findings that are, on the other hand, similar to ours [2,3,6,7,9,16,20,21,22,25,30,31,32,33].

Lee et al. argue that lower airway lesions are more common than upper airway lesions, a finding that we do not share in our case [16]. However, Woo et al. provide in their study an order of frequency of lower airway lesions not very different from that observed in our review: laryngomalacia, subglottic stenosis, pharyngeal collapse, and supraglottitis [24].

Regarding the most frequent lower airway lesions, Lee et al., as well as other publications, present extrinsic compression (67%), followed by bronchomalacia, tracheal stenosis, and mucous plug, with a high rate of pathology in the trachea (71%) [5,14,16,19,22]. This order is also not very different from that presented in our study.

Finally, in the cohort of Wu et al., there was a slight change with tracheobronchomalacia (37.3%), tracheal bronchus (34.1%), tracheobronchial stenosis (25.4%), laryngomalacia (11.4%), and subglottic stenosis (7.6%) [12]. They associate with a greater odds of airway anomaly those with premature birth, genetic syndromes, and preoperative ventilator use [12,19].

On the other hand, our data confirm a higher prevalence of airway anomalies among children with polymalformative syndromes, and especially in Down syndrome. This fact has been corroborated by other studies, such as Ghezzi et al., in which it is reflected a higher frequency of airway malformations such as tracheomalacia, tracheal bronchus and bronchomalacia, and other comorbidities associated with the syndrome, like congenital heart diseases, dysphagia, gastroesophageal reflux, musculoskeletal involvement, obesity, and immunologic impairments. For these patients, a multidisciplinary approach is imperative [34].

Fifty percent of the disease appears in the first year of life, a fact demonstrated by the average age in most published articles, and is also compatible with the predominance of studies conducted in children under one year of age in our review. However, survival has increased considerably in recent years, despite cases of difficult treatment and progression due to associated comorbidity and heart defects not corrected at the initial surgery, thanks to early diagnosis and treatment [8,18,19,20].

The mean surgical age in these cases is 8 months, with a mean weight of 8 kg, although Nayak et al. presented a cohort of 53 patients in 2012 with a mean age of 3 months and 4 kg; these data likely depend on each center’s protocol and the severity of the underlying cardiac pathology [9,28]. On the other hand, Ayten et al. showed 6 patients from 4 months to 6 years old and 33% of mortality [11]. While, in Lee et al., the mean age at which FB was performed for diagnosis was 6 months, and none suffered complications [16]. Our mean age is somewhat higher, although we agree with the absence of complications during the process [16]. The delay in performing fiberoptic bronchoscopy in newborns is probably due to the need to achieve a minimum weight before cardiovascular surgery, as fiberoptic bronchoscopy was performed after surgery in all our cases. According to complications, Castillo et al. report bleeding, fever, desaturation, bronchospasm, or pneumothorax [14].

FB contributes to a specific and definitive diagnosis in 90% of cases, and a treatable pathology is detected during the procedure in 20% of cases [10,28]. Likewise, Grohmann et al. report the possibility of combining simultaneous cardiac catheterization with bronchoscopy to take advantage of respiratory compression screening, balloon therapy, and vascular stent placement, if necessary, in the same procedure [11,18,26].

It should be noted that in our case, and despite cases reported in the literature [3,4,9], angiography or esophagogram was not performed as the first diagnostic technique; instead, bronchoscopy was used as the first line of diagnosis, avoiding the use of contrast agents. Considering that most respiratory symptoms improved and/or resolved after diagnosis with bronchoscopy, and that 23.66% were resolved in a single procedure, this could contribute to changing clinical practice by indicating early bronchoscopy in heart patients undergoing surgery.

This also opens the door to further studies to avoid late diagnosis, allowing for fiberoptic bronchoscopy to be performed even before surgery to more specifically define the severity of the findings and their correlation with morbidity, mortality, and subsequent outcome.

Based on our findings and what has been published in the literature, our proposal would be to include flexible fiberoptic bronchoscopy as a first-line diagnostic and therapeutic tool in cases of patients with congenital heart disease and respiratory complications, even taking advantage of the catheterization procedure to perform both at the same time.

## 5. Conclusions

Flexible fiberoptic bronchoscopy is a useful and rapid method for diagnosing airway malformations associated with congenital heart disease and can play a significant role in its management and prognosis. Therefore, flexible fiberoptic bronchoscopy should be considered in the initial diagnosis of patients with heart disease, as well as part of the therapeutic support, whenever they show respiratory symptoms [6].

## Figures and Tables

**Table 1:** Congenital heart defects presented by patients.

Congenital Heart Disease	*n* (%)
Valvular heart disease	21 (14.6)
Transposition of the great vessels	19 (13.2)
Atrioventricular canal defect	19 (13.2)
Ventricular septal defect	18 (12.5)
Tetralogy of Fallot	13 (9.0)
Coarctation of the aorta	13 (9.0)
Atrial septal defect	9 (6.3)
Ductus arteriosus	5 (3.5)
Truncus	5 (3.5)
Double outlet ventricle	4 (2.8)
Single ventricle	4 (2.8)
Anomalous pulmonary drainage	3 (2.8)
Hypoplastic left heart	2 (1.4)
Aortopulmonary window	1 (0.7)
Total	144

**Table 2:** Findings in fiber bronchoscopy classified according to location.

Findings *n* (% of Total Procedures)	Findings *n* (% of Total Procedures)
**UPPE** **R AIRWAY**	
Laryngotracheomalacia	43 (38)
Supra/subglottic stenosis	37 (33)
Vocal cord paralysis	18 (16)
Glottic edema	5 (4.5)
Total upper airway findings	112 (56)
**LOWER AIRWAY**	
Extrinsic compression	37 (42)
Mucus plug	23 (26)
Nonspecific airway inflammation	16 (18)
Bronchomalacia	9 (10)
Tracheobronchial fistula	2 (2.3)
Plastic bronchitis	1 (1.1)
Total lower airway findings	88 (44)

**Table 3:** Indications for which fiberoptic bronchoscopy was performed in patients with congenital heart disease in our study.

Indications for Fiberoptic Bronchoscopy	*n* (%)
Stridor	85 (42.7)
Persistent atelectasis	49 (24.6)
Intubation-extubation failure	18 (9)
Pump egress failure	14 (7)
Recurrent pneumonia	13 (6.5)
Hemoptysis	11 (5.5)
Persistent respiratory distress	9 (4.5)
Total	199

## Data Availability

Data supporting reported results can be found in the tables shared in the article.
